# Beyond Title IX and Sex-for-Marks: A Critical Review of Gender-Based Violence Scholarship in U.S. and African Higher Education

**DOI:** 10.3390/bs16101953

**Published:** 2026-10-20

**Authors:** Joyce Adeola Jekayinoluwa, Melinda Lemke

**Affiliations:** 1 Department of Global Gender and Sexuality Studies, University at Buffalo, State University of New York, Buffalo, NY 14260, USA; 2 Department of Educational Leadership and Policy, University at Buffalo, State University of New York, Buffalo, NY 14260, USA; malemke@buffalo.edu

**Keywords:** African feminism, decolonial feminism, educational policy, gender-based violence (GBV), institutional accountability, intersectional feminism, higher education, sex-for-marks, Title IX

## Abstract

While prolific research exists on campus gender-based violence (GBV), less focuses on theoretical frameworks that shape how the problem is understood and what forms of institutional response are possible. Guided by PRISMA 2020 procedures, this systematic review synthesized 52 peer-reviewed studies, between 2020 and 2025, on GBV in higher education in the United States, Nigeria, and South Africa. Findings reveal that most studies approach GBV through behavioral, psychological, or compliance-oriented frameworks, with limited research that engaged intersectional, decolonial, or African feminist perspectives. Where these frameworks are used, they illuminated dimensions of power, colonial legacies, institutional culture, and the uneven experiences of minoritized students that dominant approaches often overlook. The review also found a striking convergence across contexts: despite significant differences in policy environments, universities in both the United States and Nigeria exhibit similar patterns of underreporting, institutional mistrust, reputation management, and limited accountability. Overall, findings underscore that the marginalization of feminist theory is a research limitation that constrains how campus GBV is understood and addressed across diverse contexts. By bringing U.S. and African scholarship into conversation, this review demonstrates how theory shapes knowledge production, institutional accountability, and the possibilities for transformative responses to GBV in higher education.

## 1. Introduction

Universities are widely understood to be places of learning, equity and social transformation (Ahmed, 2012). Gender-based violence (GBV), however, remains a serious and persistent problem within their institutional structures (Ahmed, 2021). Decades of policy reform and prevention efforts have not eliminated gendered harm from higher education systems around the world, and a key reason for this persistence is that much of the existing research has not asked the right questions. Most studies approach campus GBV through behavioral or compliance-focused lenses, centering individual misconduct and institutional risk management instead of examining the deeper conditions that allow harm to continue. Mainstream institutional answers to sexual violence, including the research that informs them, are argued to serve the interests of racially and economically privileged women—thereby contributing to a politics of protection, or responses that expand institutional authority and leave the underlying conditions of harm untouched (Phipps, 2020). This dynamic is not external to universities, but constitutive of how they function: institutions adopt the language of equity while organizing themselves to resist the structural changes that equity demands (Ahmed, 2012, 2021; Mama, 2003). This is borne out empirically. In a 2025 national survey of Nigerian public universities, the leading reason students gave for not reporting sexual harassment was not fear of retaliation or of failing a course, but distrust that the university would protect them (28.6%) (Alliances for Africa, 2025).

To situate this argument, it is worth discussing forms and prevalence of campus GBV in United States and African university contexts. These figures are not directly comparable, as the underlying studies use different definitions, samples, and time frames. In the U.S., the 2019 Association of American Universities found that 25.9% of undergraduate women reported that since entering college, they experienced sexual physical force with the inability to consent. Among undergraduate women, 59.2% reported experiencing harassment, 14.1% intimate partner violence, and 10.0% stalking (Cantor et al., 2020). In Nigeria, a national survey of 18 public higher education institutions found that 31.7% of university students experienced sexual violence, including sexual harassment (25.2%), attempted rape (19.1%), and rape (10.4%), with even higher rates at polytechnics (41.5%) and colleges of education (44.4%) (Ogunfowokan et al., 2024). Additional research conducted across 4032 students and staff at 12 public universities found that 70% of female students and 30% of male students experienced at least one form of GBV on campus, with sexual misconduct, including unwanted touching, inappropriate comments, verbal abuse, and stalking, identified as the most common form (Alliances for Africa, 2025). In South Africa, a stratified random sample of 451 female students at one university found lifetime and past-year prevalence of sexual violence to be 37.9% and 25.3%, respectively (Ajayi et al., 2021), and a separate multi-campus study using a convenience sample found that 20% of 1293 female students experienced past-year partner sexual violence or non-partner rape, with higher rates at Technical and Vocational Education and Training (TVET) colleges (27%) than at universities (15%) (Machisa et al., 2021). These campus-based figures are higher than the national lifetime sexual violence prevalence of 9.8% found among South African women generally (Zungu et al., 2024), suggesting elevated risk within higher education settings specifically. Taken together, campus GBV spans harassment, coercion, stalking, intimate partner violence, and rape, and transactional exploitation such as sex-for-marks is one form among several.

Research on United States and African universities shows how institutional cultures, power structures, and deliberate silences allow harm to continue despite the existence of various policies and laws (Bondestam & Lundqvist, 2020; Dufour, 2024; B. Mapayi et al., 2023b). In the U.S., Title IX (1972) of the Educational Amendments were designed to prevent harms based on sex within primary, secondary, and postsecondary educational institutions receiving federal dollars (Pub. Law 92–318). Indeed, Title IX and a network of additional federal and state laws are tools to prevent GBV and promote prosocial behaviors among young people, yet failures to make policy procedures publicly accessible, financial constraints, limited oversight, and conservative political opposition, prompt barriers to effective enforcement (Lemke & Rogers, 2020, 2023). Additionally, research shows that U.S. universities often prioritize legal and reputational protection over supporting survivors and rely on bureaucratic procedures and neutral language that reduce liability as opposed to addressing students’ actual needs (Bedera, 2022; Gomez, 2022; Suski, 2020). African universities face similar structural problems, though the cultural and political contexts differ. Studies from Nigeria and South Africa detail deeply entrenched power imbalances between faculty and students, cultures of silence around disclosure, and administrative responses that consistently protect institutional reputation over survivor-centered accountability (Brink et al., 2022). This literature also documents sex-for-marks, or the practice in which academic grades and opportunities are exchanged for sexual access to students (B. M. Mapayi et al., 2023c; Oka et al., 2022). The fact that both contexts, governed by very different policy systems, produce the same outcomes, including underreporting, institutional mistrust, and limited accountability, suggests that campus GBV is sustained by institutional patterns that remain largely unexamined across regions.

Research on GBV in higher education proliferated over the past five years, but the field remains uneven in its theoretical grounding. Many studies rely on behavioral, program evaluation, or policy-focused approaches, while fewer engage critical frameworks that examine power, inequality, and structural conditions (Bondestam & Lundqvist, 2020; Dufour, 2024). In the United States, research has focused on Title IX processes, campus climate surveys, and measuring how often GBV occurs (Anderson et al., 2021; Byrne et al., 2021). African scholarship, while growing, has examined sexual harassment and transactional sexual economies, particularly sex-for-marks practices, within university settings (B. Mapayi et al., 2023b; Oyeoku et al., 2022). Across both contexts, comparative and international analyses are limited, and studies that explicitly draw on feminist, intersectional, or decolonial approaches to explain the broader historical and structural forces shaping campus violence are especially scarce. Thus, this systematic review aims to synthesize and compare current research on campus GBV in the United States and African contexts while also exploring how intersectional, decolonial, and African feminist frameworks are used across regions. We argue that the use of such frameworks in campus GBV research, and in turn campus responses, is a political condition that shapes which questions get asked, whose harm is recognized, and what kinds of institutional accountability are considered possible.

## 2. Theoretical Guidance

Theoretical selection influences how research problems are identified, defined, examined, and analyzed and to what end research findings transform policy and practice. This systematic review draws from inter- and trans-disciplinary, feminist policy studies research that underscores how multiple theoretical frameworks permit a more critical and nuanced way of seeing, understanding, and transforming the research phenomenon than permitted by a standalone lens (Lemke, 2019a, 2019b). The review is also situated within theoretical conversations concerning how “feminisms” in policy and related methodological research simultaneously must advance co-produced knowledge, upend legacies of colonial logics internal to Western feminist thought, and collectively work to disrupt epistemological and ontological descent into an ever-increasing authoritarian future (Lemke et al., 2025). Thus, three related but distinct feminist frameworks guided this systematic review.

The first is intersectionality, developed by Crenshaw (1989, 1991) and extended by Collins (2019). Developed as a critique of the U.S. legal system and antidiscrimination law, which failed to account for the compounded disadvantages faced by Black women, today intersectionality is a theoretical device that can interrogate overlapping and interlocking systems of oppression that shape both a person’s vulnerability to harm and their ability to access institutional support. Intersectionality is particularly useful for showing how existing institutional structures produce different outcomes for people depending on who they are and what identities they hold.

The second is decolonial theory, developed by Quijano (2000) and Mignolo (2007). Decolonial theory examines how the structures of colonial power, specifically the control of knowledge, institutions, and social hierarchies, did not disappear when formal colonialism ended. The vestiges of colonialism shape how universities are organized, what knowledge is valued, and whose experiences are centered. Decolonial feminist theory, articulated most fully by Lugones (2010), brings decolonial theory and feminism into direct conversation by arguing that intersectionality, though powerful, operates within a colonial gender system without thoroughly examining how colonialism and patriarchy work together to produce gendered harm and to determine whose harm is made (in)visible. Though the concept of feminism is contested in Indigenous studies scholarship and ways of knowing (Leigh-Osroosh & Faircloth, 2025; Tohe, 2000), it is important to note here that arguments also exist that call for decolonizing intersectionality in higher educational research and troubling how so-called “critical” social science projects maintain settler colonialism through modes of knowledge production (Arvin et al., 2013; Tuck & Yang, 2014; Pelak, 2026).

The third is African feminist traditions. African universities were historically shaped by colonial and neocolonial power, making them different from Western universities in ways that Western feminist frameworks alone cannot fully explain (Mama, 2003). Nego-feminism (Nnaemeka, 2004) centers negotiation and relational strategy in contexts where confrontational approaches carry social and institutional risk. Stiwanism (Ogundipe-Leslie, 1994) insists on women’s full structural inclusion in social and institutional transformation, distinguishing African women’s situation from both Western feminist assumptions and frameworks that subsume gender under other political priorities. Ubuntu-grounded ethics (Tamale, 2020) positions safety and accountability as collective conditions as an alternative to individual outcomes, challenging the logic that makes compliance-based institutional responses insufficient in both the Nigerian and American contexts examined here.

Overall, intersectionality maps how compounded disadvantage operates within institutions, decolonial feminist theory examines the colonial histories that produced those conditions, and African feminist frameworks attend to the specific postcolonial contexts of African higher education that the other two frameworks, developed primarily in Latin American and United States contexts, cannot fully address. The tension between these frameworks is treated as productive, and this reflects the reality that no single feminist tradition is sufficient for the kind of comparative, cross-contextual analysis this review undertakes. Finally, all three frameworks share a refusal to treat GBV as simply an individual or policy problem. Each one asks what it is about the institution that makes gendered harm possible and accountability difficult to achieve. This shared refusal is what makes them useful in comparing two different contexts that produce similar outcomes.

## 3. Theoretical Gaps and Research Purpose

Despite the analytical value of intersectional, decolonial, and African feminisms, there is a paucity of systematic reviews that examine how such theoretical orientation is used in current scholarship on GBV in higher education contexts. To begin, existing reviews tend to be United States-focused and policy-focused, engage minimally with African higher education contexts, and rarely draw on feminist theories in ways that help to explain how power shapes both harm and institutional response (McMahon et al., 2021). As a result, many systematic literature reviews treat GBV primarily as a matter of individual behavior, risk factors, or policy compliance, often failing to unpack how institutional culture and social structures produce and sustain campus violence.

Comprehensive and comparative research is also lacking. GBV occurs across higher education systems worldwide, but studies rarely bring contexts into conversation—here, the United States and Africa. In the U.S., campus responses are governed by Title IX, a formal federal regulatory framework that is criticized for uneven implementation, limited oversight, and political weaponization (Lemke & Rogers, 2023; Bedera, 2022; Ostridge, 2025). Nigerian universities operate in policy environments where formal enforcement mechanisms are developing and faculty–student power imbalances, economic dependency, and cultural stigma shape the experience of GBV and related silences (B. Mapayi et al., 2023b; Oyeoku et al., 2022).

These gaps point to the need for a systematic review that compares current research in U.S. and African contexts while also exploring how intersectional, decolonial, and African feminist frameworks are used across regions. Thus, the purpose of this review was to comparatively examine what theoretical assumptions, if any, shape institutional responses proposed by the literature, and what becomes possible when those assumptions are named and challenged. Given this focus, the review addresses the following questions:

1. How has GBV in higher education been conceptualized and studied across United States, African, and similar comparative contexts between 2020 and 2025?
2. To what extent do recent studies engage or fail to engage intersectional, decolonial, and African feminist frameworks, and what does the pattern of that engagement or absence reveal about knowledge production in the field?
3. What conceptual, methodological, and geographic gaps shape current understandings of campus safety, and how might addressing these gaps transform research and policy in United States and Nigerian higher education?

Importantly, South African scholarship was included alongside Nigerian research because South Africa offers a distinct postcolonial institutional context with a more developed formal policy architecture. This allowed the review to examine differences within African higher education without reducing the continent to a single uniform context. In doing so, this review provides insights into how institutional conditions produce structurally similar outcomes, and why those outcomes require theoretical, not purely technical, explanations.

## 4. Methodology

This systematic review followed a structured process to identify and analyze recent (2020–2025) peer-reviewed journal articles on GBV in higher education contexts. The review followed core PRISMA 2020 guidelines for systematic reviews (Page et al., 2021). Study selection process is reported using the PRISMA 2020 flow diagram for database and register searches, with no records identified from registers. The search strategy combined broad database searches with clearly defined screening and analytic steps to ensure the process was transparent and could be reproduced by researchers with access to a similar search engine.

Three searches were conducted in University at Buffalo, SUNY Libraries databases: Academic Search Complete, the Gender Watch Collection, and OneSearch, a discovery platform that also indexes the first two. Together, this permitted retrieval of interdisciplinary research across education, gender studies, law, psychology, sociology, and related fields. A record of database-specific search queries and results was maintained throughout the process. Search terms were developed, refined, and combined using Boolean operators to include sexual harassment, sexual assault, campus violence, reporting, institutional responses, prevention programs, and African feminist, intersectional, or decolonial perspectives. Filters for publication date (2020–2025), peer-reviewed status, English language, and full-text availability were applied at the search stage; OneSearch results were also limited to open-access articles.

Studies were eligible if they were empirical (qualitative, quantitative, mixed-methods, or systematic review designs) and focused on GBV within higher education settings, including universities, colleges, or international branch campuses. One aim of the review was to assess how often African feminist, decolonial, and intersectional frameworks appear in the literature, so studies did not need to use these lenses to be included. Including all relevant studies, regardless of theory, allowed the review to offer a fuller picture of the field, including research that did not engage feminist perspectives. Studies were excluded if they focused on settings outside higher education, were not empirical, were published before 2020 or after 2025, or did not meaningfully address GBV.

**Figure 1:**
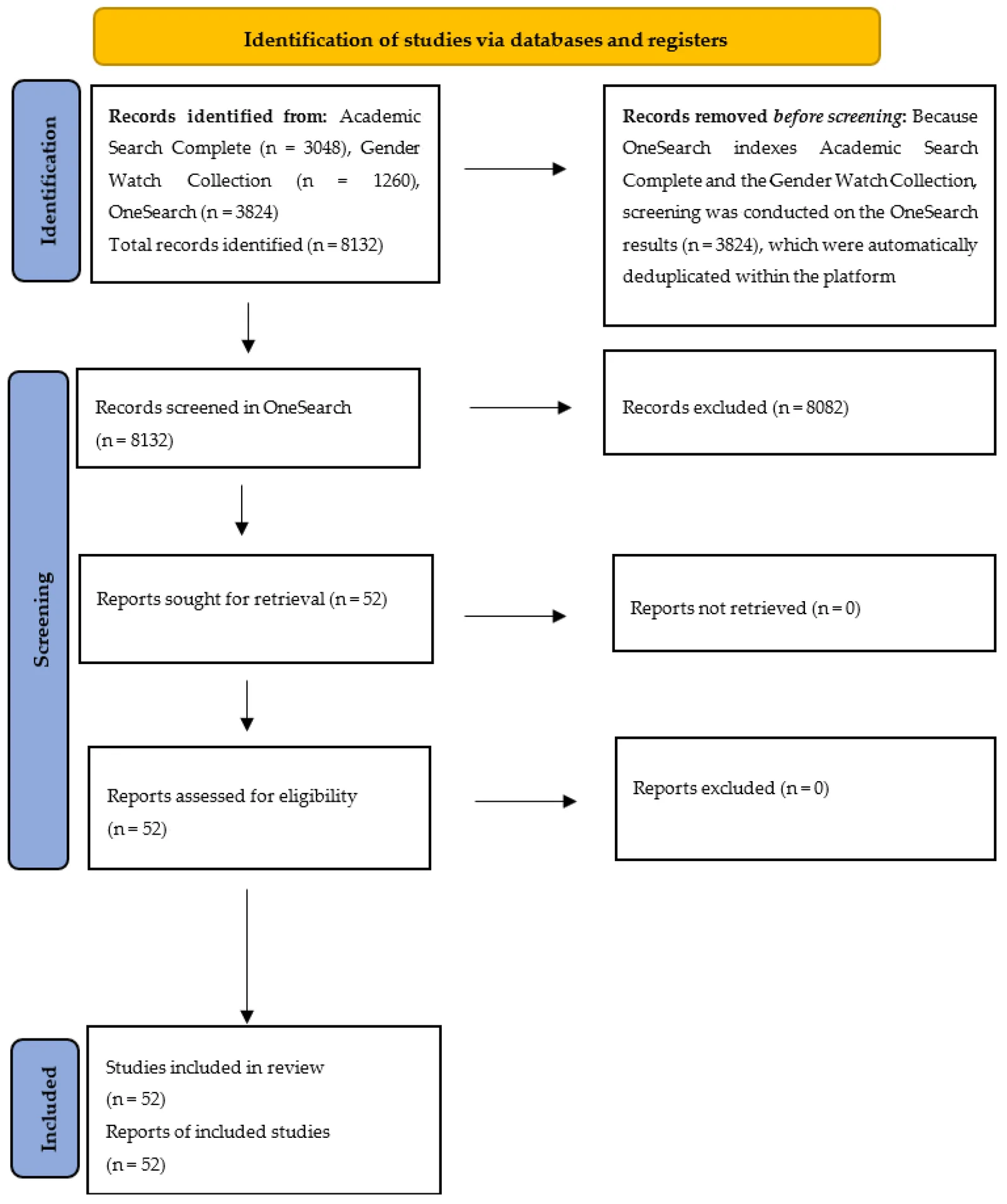
PRISMA 2020 flow diagram of study selection. Note. Adapted from Page et al. (2021). The PRISMA 2020 statement: An updated guideline for reporting systematic reviews.

As depicted in Figure 1, the database searches produced 8132 total records (Academic Search Complete, *n* = 3048; Gender Watch Collection, *n* = 1260; OneSearch, *n* = 3824). Since OneSearch indexes the other two databases, screening was conducted on the OneSearch results (*n* = 3824), which were deduplicated. The title and abstract of every record were screened for relevance to GBV in higher education, and another 3772 records were excluded. The full texts of the remaining 52 articles were then reviewed for alignment with the review aims, conceptual clarity, and methodological quality.

### Thematic Analysis

Data extraction followed structured processes including authorship and year of publication; country or region of study; population examined, such as students, faculty, or institutional actors; the form of GBV addressed; methodological approach; key findings; and the extent to which feminist theory was explicitly or implicitly engaged. Data extraction was combined with thematic feminist theoretical coding to develop an interpretive understanding of patterns in how GBV was defined, measured, and discussed across different institutional and national contexts, as well as how studies conceptualized institutional responsibility and the structural dynamics shaping GBV on campus. Although the review includes studies from multiple regions, analytical attention was focused on United States and African university contexts. Study geographic distribution reflects available peer-reviewed scholarship meeting the inclusion criteria, with African studies appearing most frequently from Nigeria and South Africa.

Thematic analysis initially produced six areas of focus that captured the breadth of the literature across regional, methodological, and theoretical dimensions. For clarity and argumentative coherence, these six areas are presented in the findings under three headings: How Campus GBV is Studied and Why it Matters; What Dominant Frameworks Cannot Explain; and What Feminist Frameworks Make Visible and Why their Absence Matters. These themes do not alter the findings but reflect a deliberate choice to present the evidence in a structure that centers the review’s theoretical argument rather than offering a purely descriptive inventory.

## 5. Findings

The database searches initially produced 8132 records. After automatic deduplication in OneSearch, 3824 records were screened by title and abstract, and 3772 were excluded. The final synthesis therefore included 52 studies published between 2020 and 2025 (see Figure 1).

## 6. How Campus GBV Is Studied and Why It Matters

The fifty-two studies included in this review differ widely in regional focus, methodology, and theoretical orientation (See Table 1). When examined together, the articles cluster around three dominant ways of understanding GBV in higher education. The first and most common approach frames GBV as an individual or behavioral problem, focusing on risk factors, attitudes, consent practices, and violence prevalence. The second frames GBV as an institutional process problem, centering policy reporting mechanisms, procedures, and compliance requirements, particularly within United States contexts governed by Title IX. A smaller body of scholarship, examined in the third findings section of this review, frames GBV as a structural problem rooted in systems of power, inequality, and institutional culture, and it is within that body of work that the most analytically developed contributions belong. These three approaches are not simply different methodological choices, but reflect different assumptions about where GBV comes from, who is responsible for it, and what kinds of responses are possible. As the analysis demonstrates, how the approaches are distributed across the literature is neither random nor politically neutral.

### 6.1. Behavioral and Individual Frameworks

The largest portion of the literature, concentrated primarily in United States and European contexts, approaches GBV through behavioral and psychological lenses. These studies focus on individual attitudes, risk factors, and communication patterns as the main objects of analysis. Quantitative studies in this cluster examine prevalence rates, rape myth acceptance, and consent communication among student populations (Anderson et al., 2021; Byrne et al., 2021; Palermo et al., 2022). Research examining university students’ perceptions of sexual violence perpetration found that recognition of coercive behavior varied significantly depending on whether physical force or psychological strategies were involved, reflecting how measurement instruments and questions shape what is counted as violence (de Oliveira et al., 2024). This variation points to a key limitation of behavioral measurement—what gets counted depends heavily on how questions are asked. Related research showed that how a study is framed affects what participants report. Students reported significantly higher rates of sexual victimization when research was explicitly presented as being about sexual assault rather than health, crime, or alcohol use (Anderson et al., 2021). This suggests that stigma and uncertainty about what counts as violence suppresses reporting when studies use less direct framing. Survey-based work on consent and sexual communication found that relying on ambiguous instead of explicit consent signals is associated with a higher risk of harm (Palermo et al., 2022). A scoping review of qualitative research on women students’ experiences of sexual violence across multiple countries found that institutional cultures, peer norms, and power dynamics consistently shaped disclosure decisions in ways that made reporting feel unsafe and institutional response feel inaccessible (Tarzia et al., 2024).

In African contexts, behavioral frameworks appear most frequently in Nigerian and South African studies documenting prevalence rates, risk exposure, and attitudes among student populations (B. Mapayi et al., 2023a; Oyeoku et al., 2022). These studies presented important descriptive data, and they share the same analytical limitation as their United States counterparts. Yet, by focusing on the individual as the unit of analysis, they make structural questions about institutional design, resource allocation, and governance accountability largely invisible. When violence is understood primarily in terms of individual risk and attitude, the institutional conditions that produce and sustain it are left unexamined.

### 6.2. Institutional and Compliance Frameworks

A second significant cluster frames GBV as an institutional process problem, focusing on how universities design and manage formal responses to sexual violence. In United States contexts organized around Title IX, research documents a consistent tension between procedural compliance and survivor-centered accountability—a tension that consistently resolves in favor of protecting the institution. Studies show that campus sexual assault policies frequently place responsibility on individuals, prioritize legal liability management, and use administrative neutrality to minimize institutional exposure as opposed to address students’ needs (Bedera, 2022; Suski, 2020). Hierarchical structures, gender-incompetent leadership, and neoliberal management practices are identified as organizational characteristics that facilitate GBV and undermine accountability across European and United States contexts (O’Connor et al., 2021). Broader systematic reviews reinforce these findings, reporting little evidence that formal policies reduce GBV prevalence or improve reporting outcomes, while underreporting and institutional mistrust remain widespread (Bondestam & Lundqvist, 2020).

In African higher education contexts, institutional response research is focused on governance environments where formal enforcement mechanisms are less developed and economic dependency, hierarchical relationships, and cultural stigma produce administrative cultures of silence. Research on South Africa demonstrated how national policy frameworks that appear progressive on paper operate within institutional cultures that consistently redirect accountability toward reputation management (Brink et al., 2022). Nigerian scholarship similarly documented entrenched power imbalances between faculty and students, sex-for-marks practices, and administrative responses organized around protecting the institution instead of supporting survivors (B. M. Mapayi et al., 2023c; Oka et al., 2022). Across both regions, research in this cluster reveals a common institutional pattern that universities respond to GBV in ways that fulfill formal obligations while preserving existing authority structures.

### 6.3. What These Frameworks Share and What They Cannot See

Behavioral and institutional compliance frameworks, for all their differences, share one foundational assumption: GBV is something institutions respond to rather than something they produce. While behavioral frameworks locate the problem in individual attitudes and communication practices, and position education and awareness as sufficient solutions, compliance frameworks locate the problem in procedural gaps and policy failures, and position better implementation and stronger oversight as sufficient solutions. Both approaches protect the institution from deeper scrutiny of its governance structures, epistemic hierarchies, resource allocation decisions, and historical constitution through racialized and gendered power relations. Research organized around these frameworks does not describe campus GBV neutrally, it actively participates in managing it, producing knowledge that universities can incorporate without being fundamentally challenged by it (Phipps, 2020). The dominance of these two frameworks in a field dedicated to understanding and eliminating gendered harm is not a coincidence of scholarly preference. It reflects the institutional conditions, including funding structures, publication incentives, and policy relevance criteria, under which campus GBV research is produced and valued. Understanding why these frameworks dominate, and what is lost as a result, requires turning to what they cannot explain.

## 7. What Dominant Frameworks Cannot Explain

Behavioral and compliance frameworks are not sufficient to explain campus GBV. Decades of prevalence research, policy reform, and prevention programming produced increasingly detailed documentation of campus GBV. They also found consistent evidence that GBV persists, underreporting is widespread, institutional responses fail survivors, and formal policy frameworks are routinely set aside in favor of protecting institutional reputations. The literature reviewed here provides substantial evidence of these patterns. This section examines three areas where dominant framework limitations become most visible—institutional responses to GBV and why said actions consistently fall short; how prevention initiatives are shaped and constrained by institutional boundaries; and why universities in the United States and Nigeria, operating under different state governance and policy systems, produce the same outcomes.

### 7.1. The Structural Function of Institutional Inadequacy

Research across United States and African contexts consistently documents institutional responses to GBV that fall short of genuine survivor-centered accountability. The explanation most often offered within the literature is implementation failure—meaning insufficient training, inadequate oversight, weak enforcement, or political interference. These are real and documented problems that do not explain why the same patterns of failure appear across institutions with different governance structures, policy architectures, and resource levels.

In the United States, organizational dependency shapes how sexual assault complaints are managed and often neutralized before reaching formal processes (Bedera, 2022). This happens not because Title IX is poorly implemented but because the institutional relationships governing academic employment, funding, and authority create structural incentives to manage complaints rather than adjudicate them. For example, Suski (2020) found that Title IX’s legal architecture places responsibility on individuals in ways that make structural accountability formally unavailable and politically difficult, and Ostridge (2025) documented how campus sexual violence policy operates through institutional arrangements that organize secrecy as a governance function rather than a lapse. Similarly, in South Africa, research found that practitioners working within formal national policy frameworks consistently encounter institutional cultures that redirect accountability toward reputation management (Brink et al., 2022). These are not failures of will or capacity; they are features of how universities are organized as institutions whose authority, resources, and legitimacy depend in part on controlling the narrative around harm rather than eliminating its conditions.

### 7.2. Prevention as Institutional Boundary-Marking

Prevention research in this review reveals a parallel pattern. Across United States, African, and European contexts, prevention efforts are most commonly delivered through mandatory training and awareness programs. Participants consistently describe these initiatives as insufficient, disconnected from lived experiences, and organized around meeting regulatory requirements rather than producing structural change (Bloom et al., 2022; McMahon et al., 2021; Wagman et al., 2022). By contrast, research on feminist education for university staff found that training grounded in feminist pedagogy, which emphasizes power, institutional responsibility, and survivor-centered accountability, produced qualitatively different outcomes than procedural or information-based approaches (C. Jones et al., 2021).

Compliance-based models persist and expand despite this evidence—and the reason is not ignorance of better alternatives. Feminist pedagogical models implicate the institution directly by demanding that universities examine their own governance, leadership composition, and resource allocation as conditions that enable GBV. Compliance models fulfill regulatory requirements without disturbing institutional authority and thus buttress a depoliticizing effect that extends to how GBV itself is named. For example, Matus et al.’s (2024) critical review of the GBV literature in higher education found that 79% of studies address gender bias rather than gender violence. Bias language locates the problem in individual attitudes and cognition, making institutional and structural conditions neutral and thus invisible as sites of accountability. What is framed as bias can be addressed through awareness training; what is named as violence implicates the conditions that allow it. In other words, the focus on bias is not a neutral descriptive preference, but a structural choice that protects institutions from the harder questions that feminist frameworks would require them to address.

### 7.3. Convergence That Demands Explanation

The most analytically significant finding across this review is also the least theorized within it. Universities in the United States and Nigeria, operating under radically different governance, policy, and social economy systems, produce structurally similar outcomes. In the United States, Title IX provides a formal federal reporting framework with legal enforcement mechanisms, institutional compliance requirements, and decades of policy development. In Nigerian universities, research documents faculty–student power imbalances, institutional accountability challenges, transactional sex, and quid pro quo sexual harassment, including sex-for-marks practices (B. Mapayi et al., 2023b; Oyeoku et al., 2022). These two contexts could not appear more different in their structural, systems, and normative architectures.

The outcomes, however, are strikingly similar, as research across both contexts documented widespread underreporting, deep institutional mistrust, administrative responses organized around reputation management, and prevention programs that remain disconnected from students’ lived realities. Research found that international students navigating United States institutional contexts face compounded barriers, including immigration concerns, language barriers, and limited trust in university authorities, that parallel the economic dependency and cultural stigma shaping disclosure decisions in Nigerian university contexts (Postel, 2020; Thomas et al., 2024). Studies examining university responses across European contexts similarly documented institutional silence as a governance function rather than an oversight failure (Pilinkaite Sotirovic et al., 2024). This convergence is too consistent and too widespread to be explained by policy gaps, implementation failures, or cultural difference.

What this convergence requires is frameworks capable of examining the institutional logic that produces these shared outcomes and that can account for how universities across different national, cultural, and policy contexts are shaped by gendered, racialized, and colonial power relations that compliance frameworks are structurally unable to interrogate. That framework is not found in the dominant literature; it appears in a small but analytically powerful cluster of studies drawing on intersectional, decolonial, and African feminist traditions, and it transforms what the evidence can mean.

## 8. What Feminist Frameworks Make Visible and Why Their Absence Matters

The studies in this cluster are drawn primarily from United States and United Kingdom contexts, with one study from Ethiopia and one from a global comparative frame. The near-complete absence of explicitly feminist scholarship from Nigerian and South African university contexts is itself among this section’s most significant findings. Eleven of the 52 studies that explicitly name a feminist lens as guiding the research design, analysis, or both are not evenly distributed by context, but are concentrated in United States and United Kingdom; they also are more likely to appear in feminist, legal, and education journals than in public health or psychology outlets (see Table 2). Feminist theory is almost entirely absent from the African scholarship in this review; thus, understanding what these studies make visible, and what their marginalization means, requires examining them not as a minor strand of the literature but as its most analytically developed core.

### 8.1. What Intersectional Frameworks Make Visible

The studies in this cluster that draw most explicitly on intersectional feminist frameworks show what becomes possible when overlapping systems of race, gender, class, and institutional power are examined together and in more detail than other studies. To begin, Newton (2025) centered Black women’s accounts of racist patriarchy in both visible and less visible campus spaces. Her analysis revealed how racist patriarchy and gender hierarchies within Black communities together shape experiences of harm, silencing, and exclusion in ways that neither race-neutral feminist frameworks nor gender-neutral antiracist frameworks can fully capture. The institution that emerges from Newton’s analysis was not one that fails Black women through oversight or poor implementation; it is one whose spatial, social, and epistemic organization actively produces their vulnerability and makes their harm invisible to the administrative gaze.

In a second study, J. Jones (2025) applied an intersectional lens to campus sexual violence disclosure across a global frame. Here, disclosure decisions were shaped not by whether reporting mechanisms exist but by the intersection of cultural expectations, shame, institutional credibility, and power imbalances that vary depending upon identity and context. This finding directly challenges a core assumption of the dominant literature—that better reporting mechanisms produce better reporting outcomes. That assumption can only hold if disclosure is treated as an individual decision rather than a structurally shaped one. While not naming intersectionality explicitly, Gomez (2022) worked through betrayal frameworks that centered the experiences of United States ethnic minority college students. Specifically, institutional betrayal compounds along racial and gendered lines. This study’s implicit rather than named intersectional engagement illustrates a broader pattern in the literature—frameworks that do intersectional work without claiming an intersectional identity make their theoretical contribution harder to build on and accumulate over time.

Finally, Kaufman et al. (2020), working in an Ethiopian university context, invoked intersectionality most explicitly in their title, examining the intersection of GBV and risky sexual behavior, though the depth of framework application across the analysis is uneven. That this partial engagement represents the closest the African scholarship in this review comes to an explicitly intersectional analysis speaks to how much research is needed to bring intersectional feminist frameworks to bear on GBV in African higher education.

### 8.2. What Decolonial Feminist Frameworks Make Visible

The most sustained application of decolonial feminist and transformative justice frameworks to campus sexual assault was found in the work of Mendez (2020). Rather than evaluating whether Michigan State University’s Title IX response to the Larry Nassar case was adequate, Mendez asked what kind of justice framework Title IX is built for and who it consistently fails to serve. This analysis keenly demonstrated that a criminal conviction and institutional settlement that followed reproduced settler-colonial and carceral logics that were never designed to protect survivors of color, nor queer or gender nonconforming survivors. Here, the concept of carceral creep is used to name how feminist demands for accountability historically expanded punitive state power in ways that harm the very communities they were meant to protect. Thus, in situating campus sexual assault within colonial histories of bodily dispossession and epistemic erasure, Mendez made visible questions that neither carceral nor compliance-oriented frameworks can formulate—whose safety is centered in institutional responses, whose testimony is structurally disbelieved, and what forms of accountability are imaginable beyond punishment and procedure?

Kennedy and McCann (2020) extended this line of analysis through a critical feminist and race-informed examination of testimony, unwantedness, and epistemic injustice in campus sexual assault. Their work foregrounds a question that the behavioral and compliance literature consistently avoids—not whether survivors report, but whether they are believed, and how the conditions of believability are organized by race, gender, and institutional power. This is an epistemic question before it is a policy question, and answering it requires frameworks capable of examining how knowledge about harm is produced, authorized, and circulated within institutions.

Finally, the epistemological dimensions of this argument are pushed furthest by McLean’s (2023) work on pedagogical and ethical challenges inherent in teaching about gender violence that challenges Global North dominance in GBV knowledge production. The analysis is among the most theoretically grounded in the entire review, drawing on intersectional feminism, decolonial theory, postcolonial feminist epistemology, and Ahmed’s (2004) affective economies to theorize a curriculum that actively decentres Global North epistemologies of GBV. Critically, for this review’s argument, McLean (2023) cited Oyèrónkẹ́ Oyěwùmí, a Nigerian feminist scholar who argues that gender was not an organizing principle in Yoruba society before British colonization imposed it. This makes McLean one of the very few studies in this review to engage African feminist epistemology as an analytic resource rather than a named gap. The analysis surfaces a tension the field rarely names—teaching about GBV within institutions that reproduce it requires working within the institution while simultaneously theorizing against its foundational assumptions. Importantly, McLean (2023) documented how GBV scholarship is dominated by Global North quantitative epistemologies, specifically behavioral economics and public health, that systematically erase survivor, practitioner, and Indigenous knowledges, a finding that directly corroborates this review’s central argument from inside the practice of feminist pedagogy itself. That this argument is made by a scholar based at the University of Sussex rather than in Nigeria is itself significant, and the next section of the analysis returns to what that geographical location reveals about who is doing this work and who is not.

### 8.3. What Feminist Pedagogical and Legal Frameworks Make Visible

Feminist praxis-oriented scholarship is known to center survivor voices as the primary epistemological resource for understanding what higher education institutions should do differently. For example, drawing on feminist epistemology and survivor testimony, Bull (2024) found that reporting parties’ perspectives on institutional GBV response reveal structural failures—institutions are organized to manage legal risk rather than support survivors, and that organizational logic is not incidental to their design but constitutive of it. Survivor knowledge, when centered, points to a fundamentally different conception of accountability than Title IX or its equivalents currently make available. Similarly, C. Jones et al. (2021) revealed what is possible through feminist education research and when university staff training is grounded in feminist pedagogy rather than procedural compliance. This study made clear that compliance training and feminist education are not the same project delivered with different emphases but are oriented toward entirely different outcomes. Compliance training aims to ensure that staff can fulfill mandatory reporting obligations and navigate institutional procedures, while feminist education aims to develop staff capacity to understand power, recognize structural conditions of harm, and center survivor accountability over institutional protection. Importantly, the former persists and expands across the literature because compliance-centric models fulfill institutional functions that feminist praxis disrupts. Del Gobbo (2022) extended this feminist analysis into legal terrain, examining how feminism, emotions, and legal imagination interact in the construction of campus sexual violence as a legal problem. The study found that the legal frameworks governing campus GBV are not neutral instruments but shaped by gendered assumptions about credibility, harm, and institutional responsibility that feminist legal theory makes visible and that compliance frameworks cannot question.

Working within prevention research, Eriksen (2021) explicitly framed bystander training as feminist-inspired leadership education. This positions prevention as collective responsibility organized around feminist principles of power and accountability. In a different vein, Matus et al. (2024) applied a queer feminist studies of science framework to distinguish gender bias from gender violence in higher education literature. Their central finding, that 79% of studies address gender bias rather than gender violence, is itself a sustained feminist argument—the field’s terminological preference depoliticizes harm by locating it in individual cognition.

### 8.4. Why Feminist Frameworks Remain Marginal

Across these 11 studies, a consistent pattern emerged—when feminist frameworks are applied, they do not simply add nuance to existing findings, but instead they reframe the question entirely. Such lenses shift the analytical focus from individual behavior and institutional procedure to structural power, epistemic authority, and the conditions under which certain harms become visible while others do not. That this reframing appears in roughly one in five studies, and that it is almost entirely absent from the African scholarship in this review, is not incidental.

The near-complete absence of explicitly feminist frameworks from studies conducted in Nigerian, South African, and Ethiopian universities is significant. African feminist traditions, including nego-feminism (Nnaemeka, 2004), Stiwanism (Ogundipe-Leslie, 1994), and Ubuntu-grounded relational ethics (Tamale, 2020), offer conceptual tools developed within and for the postcolonial institutional contexts where this research is situated. These frameworks center negotiation, relational accountability, communal ethics, and postcolonial power in ways that neither Western behavioral models nor Western feminist frameworks fully capture. Their absence from the empirical scholarship is not explained by their unavailability or irrelevance. It reflects the epistemic hierarchies that determine which theoretical traditions are recognized as rigorous, which journals are considered authoritative, and which institutional contexts are treated as broadly applicable rather than context-specific.

This is where this review’s central concern lands most concretely—the marginalization of intersectional, decolonial, and African feminist frameworks from campus GBV scholarship is not a gap that additional, more broad, or more inclusive bibliographies can resolve; it is a structural condition of knowledge production in a field whose dominant frameworks serve the same institutional logics they are meant to study. The 11 studies examined in this section do not represent a corrective supplement to the literature. They represent a fundamentally different theory of the problem—one that asks not how institutions manage GBV but why they are structured to reproduce it. This is the penultimate question that the field will need to address if scholarship on campus safety is to produce something other than increasingly sophisticated documentation of persistent harm.

## 9. Discussion

The pattern documented across the 52 studies examined in this systematic review demonstrates that the underuse of intersectional, decolonial, and African feminisms is consistent, geographically organized, and aligned with identifiable institutional interests. The findings also revealed what the literature has, however unintentionally, been organized to protect from scrutiny. This discussion underscores the costs of theoretical sidelining for the field, key takeaways across otherwise divergent contexts, and changes needed in research and practice.

### 9.1. What Title IX and Sex-for-Marks Reveal Together

This review’s comparative focus on the United States and Nigeria was motivated in part by how different these two contexts appear. They have different governance structures, policy systems, and historical sociocultural contexts for understanding gendered harm. Title IX is one of the most formalized regulatory frameworks for addressing gender violence in the world—it is federally mandated, institutionally embedded, and extensively litigated—and yet GBV proliferates. Sex-for-marks is one of the most openly documented forms of faculty–student coercion in Nigerian higher education, and it operates largely outside formal accountability structures.

Their shared features are more striking than their differences. Both are sustained by institutional logics that prioritize protecting authority over supporting survivors. Title IX’s proceduralism neutralizes complaints, places responsibility on individuals, and insulates institutional power from structural scrutiny (Bedera, 2022; Ostridge, 2025; Suski, 2020). Sex-for-marks operates within hierarchical structures where economic vulnerability, academic gatekeeping, unequal faculty–student power relations, and stigma around disclosure can constrain reporting and institutional accountability (B. Mapayi et al., 2023b; Oka et al., 2022; Oyeoku et al., 2022). One context has an extensive formal federal policy with decades of legal development behind it. The other has no equivalent national legal framework, making institutional accountability dependent on discretion, cultural norms, and developing enforcement capacity that remains uneven across institutions.

This systematic review placed Title IX and sex-for-marks in direct conversation, demonstrating that the problem is not the presence or absence of formal policy but the institutional logic that formal policy, in both contexts, is made to serve. That logic cannot be named, let alone challenged, by behavioral or compliance-oriented frameworks. It can only be named by frameworks willing to ask who universities are built for, whose harm they are designed to manage, and whose knowledge about that harm they are structured to exclude.

### 9.2. Implications for Research and Policy

The patterns identified in this systematic review carry different but related implications for research and policy in the United States and Nigeria. In the United States, the concentration of scholarship around Title IX processes generated important insights into procedural harm and institutional betrayal. At the same time, this concentration has narrowed what the field can see and ask. Future research would benefit from moving beyond compliance evaluation toward examining how governance structures, policymaking, leadership composition, resource allocation, and epistemic authority shape both the prevalence of GBV and the conditions of institutional accountability. Intersectional feminist frameworks are particularly important here. Research that does not account for the ways that sexual orientation, race, nationality, class, and ability, among other identity markers, shape students’ experiences of both harm and institutional response cannot produce policy recommendations that serve the full range of students Title IX is intended to protect.

In Nigerian higher education, existing scholarship has effectively documented lecturer–student power imbalances, sex-for-marks practices, and cultures of silence. The critical next step is deeper theorization, specifically through decolonial feminist and African feminist analytic traditions that can situate these dynamics within their historical, political, and institutional contexts. Without sustained engagement with frameworks that examine how colonial legacies shape university governance, authority structures, and the conditions of women’s academic participation, GBV in Nigerian universities risks being framed as a cultural and localized institutional problem requiring attitudinal change, as opposed to gendered harm rooted in structural violence.

For both contexts, the most significant research gap is theoretical. The field needs scholarship that treats the absence of feminist frameworks not as an oversight to correct but as a phenomenon to explain, one that raises questions about who produces knowledge about campus GBV, under what institutional conditions, and in whose interests. Comparative research that brings contexts into direct dialogue, as this review did with the United States and Nigeria, is particularly well positioned to make that argument. The convergence of outcomes across such different institutional contexts is itself the most powerful evidence that the explanation must be structural rather than situational.

This structural convergence still leaves unresolved why these frameworks remain marginal in the first place, a question this review’s design cannot settle, since it documents patterns in published scholarship rather than the decisions behind them. The pattern is consistent with three explanations the broader literature on African feminist knowledge production suggests, and which likely operate together. The first is methodological hierarchy: Western academic traditions long have treated quantitative, positivist approaches as more rigorous than feminist, qualitative, and community-based research (Falola & Yacob-Haliso, 2017), and behavioral and compliance-focused study designs offer few conventions for engaging feminist theory. The second is institutional and political interest: African feminist scholarship has faced sustained pressure to justify its legitimacy against nationalist and traditionalist backlash, which diverts energy from developing its fuller political philosophy (Salami, 2025), and research that names governance and institutional authority as sources of harm is more difficult to fund and bring into policy processes (Phipps, 2020). The third is the colonial structuring of academic knowledge itself: African universities continue to privilege Western theoretical and administrative models over Indigenous frameworks (Tamale, 2020), and scholars working in African feminist and gender studies continue to face structural barriers within the institutions they seek to transform (Baderoon et al., 2024). These explanations reinforce one another—how much weight each carries is an empirical question to be explored through interviews with scholars, editors, and reviewers, as well as analysis of funding and curricula.

## 10. Conclusions

This systematic review began with a puzzle—two higher education systems that look almost nothing alike producing outcomes that look remarkably similar. The institutional and policy differences between the United States and Nigeria are real and consequential. Yet, persistent underreporting, institutional mistrust, administrative responses organized around reputation management, and prevention programs disconnected from students’ lived realities appear in both contexts with a consistency for which no single policy framework or cultural explanation can account. This review systematically explains that persistence, and that explanation implicates both systems.

The arguments advanced in this review are not new—scholars have long documented how universities adopt the language of equity while resisting the structural transformations that equity would require (Ahmed, 2004; Mama, 2003; Phipps, 2020). The contribution this review makes to that argument is an empirical map of exactly how those dynamics operate across 52 peer-reviewed empirical studies, representing two different higher education contexts, and within a field that presents itself as dedicated to eliminating gendered-raced harm. When the same outcomes appear in Nigeria and the United States, the explanation cannot be found in national policy failure or cultural resistance to change. It must be found in the institutional logics that organize universities across different societies to manage gendered harm, and in the research frameworks that, by staying within the behavioral and compliance-oriented mainstream, produce knowledge that serves those logics.

The comparative method this review employed is itself an argument, not merely a design choice. Placing Title IX and sex-for-marks in dialogue does something that no single-country study can do—it makes visible that the underuse of feminist theory takes a specific and different form in each context. In the United States, feminist frameworks coexist with the dominant literature without challenging it, present at the margins of a field organized around compliance infrastructure and behavioral measurement. In Nigeria, the lenses most specifically suited to analyzing the conditions Nigerian scholarship documents are almost entirely absent from that scholarship, while scholars working outside Africa engage African feminist epistemology more substantively than those working within it. Placing those patterns side-by-side produces an argument that the distribution of frameworks across the field is not random but organized, and that organizing principle is the same institutional logic the frameworks themselves are best equipped to interrogate. Comparative cross-regional research is therefore not a methodological supplement to the field, it is a necessary condition for producing explanations that match the problem scale.

The stakes of these gaps extend beyond the academy. A field that continues to measure success by compliance metrics will continue to produce findings that universities can absorb without changing structures that reproduce harm. A field that evaluates prevention by attitude change will fail at oversight around the governance structures, leadership cultures, and resource allocation decisions that make harm structurally possible. A field that draws policy recommendations for Nigerian universities from research that documents GBV without theorizing its conditions will reproduce interventions organized around Western institutional assumptions that do not fit the contexts they are meant to address. On the question of whether the scholarship produced in this field is capable of serving the people whose experiences it documents, the literature reviewed here, across both contexts, falls consistently short.

Research on GBV in higher education is at a crossroads. Scholarship produced an extraordinary corpus on the persistence of gendered harm in higher education, but far less exists on why this permanency is consistent, cross-contextual, and resistant to interventions the field designed to address it. The answer that this review proposes is not a call for more research or better policies—simply put, the field needs to ask a different kind of question entirely, one that treats the institution not as the site of GBV response but as the site of GBV reproduction. The contribution of this review to that project, and to the field, is to demonstrate that a systematic review, a methodology typically associated with evidence-based policy and behavioral research, can be turned toward a structural feminist argument without losing its rigor. It can map not only what the literature finds but what the literature is organized to protect from examination. That, too, is empirical work, and it points toward a research agenda wherein framework silencing is as analytically significant as their presence, and in which the question worth pursuing is not how universities manage GBV, but why they are built to reproduce it, and what it would mean to build institutions that are not.

## Figures and Tables

**Table 1:** Feminist Theoretical Framework Engagement. Framework Engagement Levels Across 52 Studies on Gender-Based Violence in U.S. and African Higher Education (2020–2025).

Level of Engagement with Feminist Theory	Studies
Central	Bull (2024); Del Gobbo (2022); C. Jones et al. (2021); J. Jones (2025); Kennedy and McCann (2020); Matus et al. (2024); McLean (2023); Mendez (2020); O’Connor et al. (2021); Ostridge (2025); Newton (2025)
Partial-Central	Eriksen (2021)
Partial	Gomez (2022); Freude et al. (2024); Oyeoku et al. (2022)
Partial-Implicit	Abu-Odeh et al. (2020); Brink et al. (2022); Goicolea et al. (2022); Oka et al. (2022); Vieira et al. (2023); Kaufman et al. (2020)
Implicit	Bovill et al. (2022); Pilinkaite Sotirovic et al. (2024); Bovill and Podpadec (2023); Postel (2020); Tarzia et al. (2024); Bedera (2022); Dufour (2024); Emelianchik-Key et al. (2023); Cai et al. (2021); Wagman et al. (2022); Bondestam and Lundqvist (2020); Bloom et al. (2022); Orchowski et al. (2020); Bloom et al. (2021); Palermo et al. (2022); Ruiz-Eugenio et al. (2020); Adams (2021); McMahon et al. (2021); Suski (2020)
Absent	Thomas et al. (2024); Hendricks and Kanjiri (2020); Anderson et al. (2021); Anderson et al. (2022); Hendricks (2022); B. Mapayi et al. (2023a) [x3]; Chambers et al. (2021); Banda-Chitsamatanga and Ntlama (2020); Byrne et al. (2021); de Oliveira et al. (2024)

Note. Engagement level definitions include the following: Central or feminist framework explicitly named and applied as primary analytic lens throughout study (11 studies); Partial-Central or feminist framework named and substantively applied, but quantitative methodology limits full operationalization (1 study); Partial or feminist framework named, but applied unevenly (3 studies); Partial-Implicit or feminist framework named in a limited way without sustained application (6 studies); Implicit or feminist framework concepts engaged without naming formal framework (19 studies); Absent or no feminist framework engagement (12 studies).

**Table 2:** Feminist Theoretical Framework as Central. Feminist Theory Centered in 11 Studies on Gender-Based Violence in U.S. and African Higher Education (2020–2025).

Study	Feminist Framework	Framework Type
Bull (2024)	Feminist epistemology; transformative justice (Ahmed)	Feminist qualitative; survivor voice
Del Gobbo (2022)	Feminist legal theory; carceral feminism (Ahmed; Brown; Butler; Lorde)	Feminist legal theory
J. Jones (2025)	Intersectional feminism (Ahmed; Crenshaw; Collins)	Intersectional feminist
C. Jones et al. (2021)	Feminist pedagogy; feminist praxis (Belenky; Crenshaw; Collins)	Feminist pedagogy; qualitative evaluation
Kennedy and McCann (2020)	Critical race feminism (Ahmed; Crenshaw; Gilmore)	Critical race feminism
Matus et al. (2024)	Critical feminist scoping review; Queer feminist studies of science	Critical feminist; queer feminist
McLean (2023)	Affective economy (Ahmed); Decolonial theory (Lugones; Mignolo; Mohanty; Spivak); feminist pedagogy (hooks; Freire); intersectional feminism (Crenshaw; Collins); postcolonial feminist epistemology (Oyewumi)	Decolonial; feminist pedagogy; reflective practitioner
Mendez (2020)	Decolonial feminism (Lugones); carceral creep (Kim); transformative justice	Decolonial feminist theory and praxis
Newton (2025)	Critical race feminism; Black feminist thought; intersectional ethnography	Critical race feminist ethnography
O’Connor et al. (2021)	Feminist organizational theory (Acker; Crenshaw; Lukes)	Feminist organizational theory
Ostridge (2025)	Feminist institutional sociology (Ahmed; Smith)	Feminist institutional sociology

## Data Availability

No new data were created or analyzed in this study. The review is based entirely on published peer-reviewed literature.
